# Natural course of fat necrosis after breast reconstruction: a 10-year follow-up study

**DOI:** 10.1186/s12885-021-07881-x

**Published:** 2021-02-16

**Authors:** Jeeyeon Lee, Ho Yong Park, Wan Wook Kim, Jeong Ju Lee, Hee Jung Keum, Jung Dug Yang, Jeong Woo Lee, Joon Seok Lee, Jin Hyang Jung

**Affiliations:** 1 Department of Surgery, School of Medicine, Kyungpook National University, Kyungpook National University Chilgok Hospital, Hoguk-ro 807, Buk-gu, Daegu, 41404 Republic of Korea; 2 Department of Surgery, Boonhongbitzro Hospital, Daegu, Republic of Korea; 3 Department of Plastic and Reconstructive Surgery, School of Medicine, Kyungpook National University, Kyungpook National University Chilgok Hospital, Daegu, Republic of Korea; 4 Department of Plastic and Reconstructive Surgery, School of Medicine, Kyungpook National University, Kyungpook National University Hospital, Daegu, Republic of Korea

**Keywords:** Breast, Carcinoma, Breast reconstruction, Fat necrosis

## Abstract

**Background:**

Although fat necrosis is a minor postoperative complication after breast reconstruction, occasionally it mimics to tumor recurrence in patients with breast cancer. Therefore, the surgeon should distinguish between benign fat necrosis and true local recurrence. The authors evaluated the clinical characteristics of fat necrosis after breast reconstruction and investigated the natural course of fat necrosis.

**Methods:**

Between 2007 and 2013, a total of 362 patients underwent breast reconstruction after partial or total mastectomy for breast cancer in Kyungpook National University Hospital. Clinicopathologic characteristics and the occurrence of fat necrosis were assessed during surveillance for 10 years of mean follow-up period.

**Results:**

There were 42 cases (11.6%) of fat necrosis after breast reconstruction with partial or total mastectomy which were confirmed by needle or excision biopsy. The fat necrosis was resolved after a mean period of 45.9 months (SD, ± 42.1) and 26 cases (61.9%) of fat necrosis were almost completely resolved (less than 5 mm) during 10-year follow-up period.

**Conclusion:**

Based on the natural course of fat necrosis, the fat necrosis after breast reconstruction can be only monitored, if pathologic confirmation was done. More than half of the cases will be resolved within 2–3 years.

**Supplementary Information:**

The online version contains supplementary material available at 10.1186/s12885-021-07881-x.

## Introduction

Nowadays, the breast surgery for breast cancer should secure not only a oncologic safety but also a better cosmetic outcome [1]. Breast reconstruction after partial or total mastectomy can be achieved with volume displacement, volume replacement techniques or implant-based surgery. The volume displacement technique is performed with the advancement, transposition, or reshaping of the breast parenchyma, whereas the volume replacement technique is performed with adjacent or distant flap surgery [2–6]. As the blood supply of the flap is supported by donor site vessels only, fat necrosis is more common with the volume replacement technique than with conventional breast-conserving surgery or the volume displacement technique.

Fat necrosis is a benign inflammatory process that is usually associated with various types of traumas including surgery, radiotherapy, biopsy, or infection [7]. Surgical intervention and additional radiotherapy for breast cancer may possibly have some effect on the incidence of fat necrosis [8, 9]. The incidence of fat necrosis after flap using breast reconstruction has been reported as approximately 4–25% of patients treated with breast-conserving surgery and adjuvant radiotherapy for breast cancer [10–12]. Fat necrosis usually occurs in the focal area that has poor blood supply, such as the peripheral site of the reconstructed flap. Therefore, the incidence of fat necrosis is higher when the volume replacement technique is used instead of the volume displacement technique for breast reconstruction.

Fat necrosis may be suggested with typical imaging features (calcified nodule in mammography and ultrasonography, peripheral site of the reconstructed flap, and poor vascular supply) [13]. However, when the shape of the necrotic nodule shows an irregular or spiculated margin with suspicious microcalcification, it is necessary to distinguish it from tumor recurrence with pathologic confirmation [14].

Although fat necrosis is a minor complication after breast reconstruction, it may cause patient anxiety and inconvenience and sometimes mimic tumor recurrence on the ipsilateral breast. Therefore, it is important for surgeons to understand the natural course of fat necrosis. This study evaluated the clinical characteristics of fat necrosis after breast reconstruction and investigated the natural course of fat necrosis.

## Methods

From 2007 to 2013, a total of 362 patients underwent breast reconstruction after partial or total mastectomy for breast cancer in Kyungpook National University Hospital.

The treatment strategy for breast cancer was determined by multidisciplinary team discussion. Multidisciplinary team discussion involved breast and plastic surgeons, oncologists, radiologists, pathologists, radiation oncologists, and nurses. Neoadjuvant chemotherapy was performed for locally advanced breast cancer (>stage IIIA). Adjuvant treatment, including chemotherapy, radiotherapy, hormone treatment, or target therapy, was performed postoperatively if considered necessary.

Breast cancer tissues were removed completely with a safe margin of > 2 mm, and the surgical margins were pathologically evaluated with frozen and permanent biopsies for the presence of tumor cells. In addition, either sentinel node biopsy or axillary lymph node dissection was performed according to the axillary lymph node status. Volume displacement or replacement techniques were individualized according to the excised breast volume and tumor location, and these techniques included reduction mammoplasty or the use of the lateral thoracodorsal (LTD) flap, intercostal artery perforator (ICAP) flap, thoracodorsal artery perforator (TDAP) flap, thoraco-epigastric (TE) flap, latissimus dorsi (LD) myocutaneous flap, transversus rectus abdominis myocutaneous (TRAM) flap, deep inferior epigastric (DIEP) flap and several other flaps. To reduce the incidence of fat necrosis and maintain vascularity, the plastic surgeons checked the doppler ultrasound on 3–4 points of skin paddle serially from intraoperative, immediate postoperative to postoperative periods. And the intact vascularity of obtained flap was defined when fresh pin-point bleeding was detected in distal margin of flap. After surgery, the plastic surgeons performed a blenching test for 2–3 s in skin flap and applied the medical leech therapy for 3 days to increase vascularity and flap survival when the blenching test result showed < 1 s and congestive status of flap.

Adjuvant radiotherapy was delivered to the ipsilateral breast with a radiation dose of 50.4 Gy in 28 fractions, and a dose of 10 Gy in 5 fractions was added to the tumor bed. When the closest resection margin was less than 0.1 cm, the patient received additional radiation (14 Gy in 7 fractions).

Clinicopathologic characteristics including age, body mass index, underlying disease, clinical and pathologic tumor size, axillary lymph node status, tumor subtype, neoadjuvant or adjuvant treatment, type of breast reconstruction, weight of the excised specimen, operative time, duration of hospital stay, and occurrence of fat necrosis were assessed. We performed the surveillance for breast cancer per 6 months for first 2 years and per 1 year for next 3 years with mammography, ultrasonography, chest X-ray and laboratory findings including CA15–3, CEA. The oncologic outcomes (loco-regional recurrence, distant metastasis, and death) during the follow-up period were evaluated.

### Follow-up of fat necrosis

When the fat necrosis is suspected in any imaging modalities or patient has clinical symptoms, we performed additional ultrasound and evaluated whether it is benign or suspicious finding. If the suspicious nodule was detected, we performed cytology, needle biopsy or excision case-by-case.

After fat necrosis was pathologically confirmed with cytology, needle biopsy, or excision biopsy, the initial detection time and duration, imaging findings, and size of the necrotic mass during the follow-up period were collected retrospectively. The clinicopathologic and operative variables of patients who had fat necrosis after breast reconstruction with breast reconstruction were also assessed. And the resolution of fat necrosis was defined when the nodule diagnosed as fat necrosis was disappeared in image work-up.

### Statistical analysis

Statistical analysis was performed using SPSS ver. 12.0 (SPSS, Chicago, IL, USA). Categorical variables were analyzed using the Χ^2^ test in univariate analysis, and oncologic outcomes were assessed by multivariate analysis using logistic regression to identify factors affecting the locoregional recurrence or distant metastasis of breast cancer. *p* values of < 0.05 were considered as statistically significant.

## Results

The mean age of patients with breast cancer who underwent breast reconstruction after partial or total mastectomy was 45.5 years (SD, ± 7.9), and the mean BMI was 23.1 kg/m^2^ (SD, ± 3.0). There were 43 patients (11.8%) with hypertension and 39 patients (10.8%) with diabetes mellitus. The mean clinical and pathologic tumor size was 2.8 cm and 2.3 cm (SD, ± 1.6), respectively, and 79 patients had axillary lymph nodes metastasis. During the 97.3 months (SD, ± 23.5) of the follow-up period, there were 22 cases (6.1%) of locoregional recurrence, 15 cases (4.1%) of distant metastasis, and 9 cases (2.5%) of death.

Among a total of 362 cases of breast reconstruction, partial mastectomy with reconstruction was performed in 123 cases (34.0%), and nipple- or skin-sparing mastectomy with reconstruction was performed in 239 cases (66.0%). The types of breast reconstruction included reduction mammoplasty or the use of the thoraco-epigastric (TE) flap, lateral thoracodorsal (LTD) flap, thoracodorsal artery perforator (TDAP) flap, intercostal artery perforator (ICAP) flap, conventional or extended latissimus dorsi (LD) myocutaneous flap, pedicled or free transversus rectus abdominis myocutaneous (TRAM) flap, deep inferior epigastric (DIEP) flap and several other flaps. The mean weight of the surgical specimen was 231.4 g (SD, ± 159.0), and the mean operative time was 458.3 min (SD, ± 116.1). The mean hospital stay was 16.1 days (SD, ± 4.5). The type of breast surgery or reconstructive surgery, weight of the removed specimen, and operative time were not associated with the occurrence of fat necrosis (Table 1).

**Table 1:** Clinicopathologic characteristics of patients who underwent breast reconstruction for breast cancer

Variables	Oncoplastic surgery (*n* = 362)	Fat necrosis	
		*n* = 42	*p* value
Mean age (years, ± SD)	45.5 ± 7.9	46.1 ± 7.4	0.328
Mean body mass index (kg/m^2^, ± SD)	23.1 ± 3.0	23.2 ± 2.1	0.311
Underlying disease (n, %)			0.402
Hypertension	43 (11.8)	3 (7.1)	
Diabetes mellitus	39 (10.8)	4 (9.5)	
Mean clinical tumor size (cm, ± SD)	2.8 ± 1.6	2.1 ± 0.6	0.086
Mean pathologic tumor size (cm, ± SD)	2.3 ± 1.6	2.7 ± 1.9	0.619
Estrogen receptor, positive (n, %)	189 (52.2)	23 (54.8)	0.709
Progesterone receptor, positive (n, %)	165 (45.6)	18 (42.9)	0.461
c-erbB2 gene, positive (n, %)	69 (19.1)	7 (16.7)	0.523
Triple-negative breast cancer (n, %)	43 (11.9)	1 (2.4)	0.098
Type of breast surgery (n, %)			0.269
Partial mastectomy	123 (34.0)	10 (23.8)	
Nipple- or skin-sparing mastectomy	239 (66.0)	32 (76.2)	
Type of reconstructive surgery (n, %)			0.222
Reduction mammoplasty	27 (7.5)	2 (4.8)	
Thoraco-epigastric (TE) flap	4 (1.1)	–	
Lateral thoracodorsal (LTD) flap	20 (5.5)	–	
Thoracodorsal artery perforator (TDAP) flap	15 (4.1)	2 (4.8)	
Intercostal artery perforator (ICAP) flap	24 (6.6)	2 (4.8)	
Latissimus dorsi (LD) myocutaneous flap	73 (20.2)	3 (7.1)	
Extended LD flap	102 (28.2)	4 (9.5)	
Pedicled Transversus rectus abdominis myocutaneous (TRAM) flap	59 (16.3)	24 (57.2)	
Free TRAM flap	29 (8.0)	4 (9.5)	
Deep inferior epigastric perforators (DIEP) flap	1 (0.3)	–	
Other flaps	6 (1.7)	1 (2.4)	
Mean weight of specimen (g, ±SD)	231.4 ± 159.0	189.1 ± 110.4	0.142
Mean operation time (minutes, ±SD)	458.3 ± 116.1	431.3 ± 106.5	0.610
Mean hospital stay (days, ±SD)	16.1 ± 4.5	16.4 ± 3.3	0.562
Neoadjuvant chemotherapy (n, %)	46 (12.7)	2 (4.8)	0.044
Adjuvant chemotherapy (n, %)	173 (47.8)	10 (23.8)	0.944
Adjuvant radiotherapy (n, %)	105 (29.0)	10 (23.8)	0.919
Mean follow-up period (months, ± SD)	97.3 ± 23.5	117.0 ± 26.9	0.184
Locoregional recurrence (n, %)	22 (6.1)	2 (4.8)	0.465
Distant metastasis (n, %)	15 (4.1)	4 (9.5)	0.799
Death (n, %)	9 (2.5)	2 (4.8)	0.625

Although 42 cases (11.6%) of fat necrosis were diagnosed during the follow-up period, there was no significant factor associated with the occurrence of fat necrosis after breast reconstruction except for the performance of neoadjuvant chemotherapy (*p* = 0.044). One patient was not received further evaluation after 30 months from surgery. Adjuvant radiotherapy were performed to ten patients (23.8%); after breast conserving surgery (*n* = 9) and mastectomy (*n* = 1).

Although there were two cases of locoregional recurrence among them, the recurred site was not breast but the axillary lymph nodes and supraclavicular lymph nodes. In multivariate analysis, it did not show any statistical significance. In addition, the incidence of fat necrosis after breast reconstruction was higher with the pedicled TRAM flap (*n* = 24, 57.2%). There were 30 cases of microsurgical flaps including free TRAM, DIEP flap and only four cases (9.5%)of fat necrosis were identified during follow up period.

**Fig. 1:**
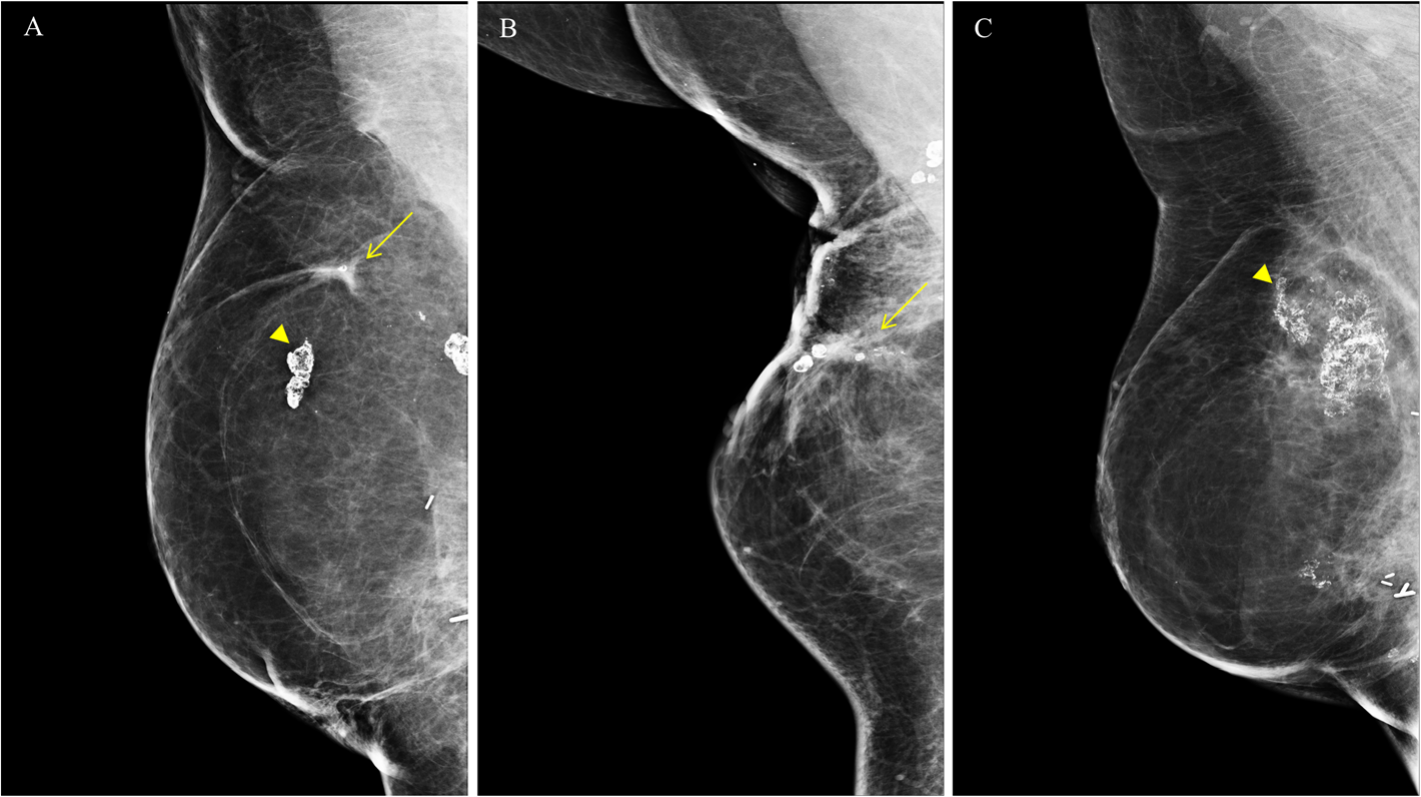
**a**-**c** Mammographic findings of fat necrosis after breast reconstruction. Dystrophic calcification (arrow heads) and architectural distortion (arrows) are common findings in the mammography of fat necrosis

**Fig. 2:**
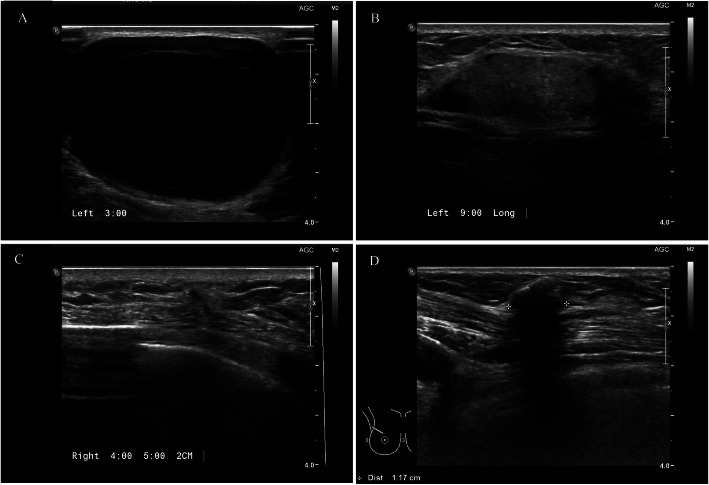
Ultrasonographic findings of fat necrosis after breast reconstruction. **a**, **b** A simple or fat-containing complex cyst is a typical ultrasonographic finding of fat necrosis. **c**, **d** Sometimes the fat necrosis mimics tumor recurrence in ultrasonography as a hypoechoic nodule with irregular margins. In these cases, pathologic confirmation is necessary to distinguish it from tumor recurrence

The pathologic confirmation of fat necrosis was obtained with fine-needle aspiration cytology (*n* = 1, 2.4%), needle biopsy (*n* = 34, 81.0%), and excision biopsy (*n* = 7, 16.7%). The mean period until the detection of fat necrosis was 21.1 months (SD, ± 17.2), and the mean size of fat necrosis was 2.5 cm (SD, ± 1.5). Most cases were detected through imaging modalities including mammography (*n* = 13, 31%) and ultrasonography (*n* = 41, 97.6%), and only one case was detected by breast magnetic resonance (MR) imaging (n = 1, 2.4%). The combined symptom with fat necrosis were nodular lesion (*n* = 3), pain (*n* = 6) and they were received surgical intervention, if patient wanted (Table 2). Although the mammographic findings of fat necrosis were typically calcification or architectural distortion with calcification (Fig. 1), ultrasonographic findings showed various shapes. Eight cases mimicked tumor recurrence, which required pathologic confirmation, and other cases involved a simple or complex cystic lesion (Fig. 2). And the pathologic results of suspicious nodules mimicking tumor recurrence are described on Table 3.

**Table 2:** Clinical characteristics associated with fat necrosis after breast reconstruction

	Fat necrosis (*n* = 42)
Mean period until the detection of fat necrosis (months, ±SD)	21.1 ± 17.2
Mean mass size of fat necrosis at the time of diagnosis (cm, ±SD)	2.5 ± 1.5
Grade (n, %)	
1 (Radiologic evidence only)	33 (78.6)
2 (Palpable but not visible)	–
3 (Palpable and visible)	3 (7.1)
4 (Painful fat necrosis)	6 (14.3)
Imaging modalities^a^ (n, %)	
Mammography	13 (31.0)
Ultrasonography	41 (97.6)
Breast MR imaging	1 (2.4)
Pathologic confirmation (n, %)	
Fine-needle aspiration cytology	1 (2.4)
Needle biopsy	34 (81.0)
Excision biopsy	7 (16.7)
Resolved status (n, %)	
Not resolved	16 (38.1)
Resolved	26 (61.9)
Mean period for the complete resolution of fat necrosis (months, ±SD)	45.9 ± 42.1

^a^Method of image modality for detection of fat necrosis could be duplicated

**Table 3:** Management of suspicious nodules after breast cancer surgery which is mimicking the tumor recurrence

	n (%)
Suspicious nodule mimicking the tumor recurrence	8 (19.0)
Confirmation method	
Needle biopsy	6 (14.3)
Excision	2 (4.8)
Pathologic results of biopsy	
Chronic inflammation with fat necrosis	3 (7.3)
Stromal fibrosis with fat necrosis	3 (7.3)
Fat necrosis with calcification	1 (2.4)
Only fat necrosis	1 (2.4)

**Fig. 3:**
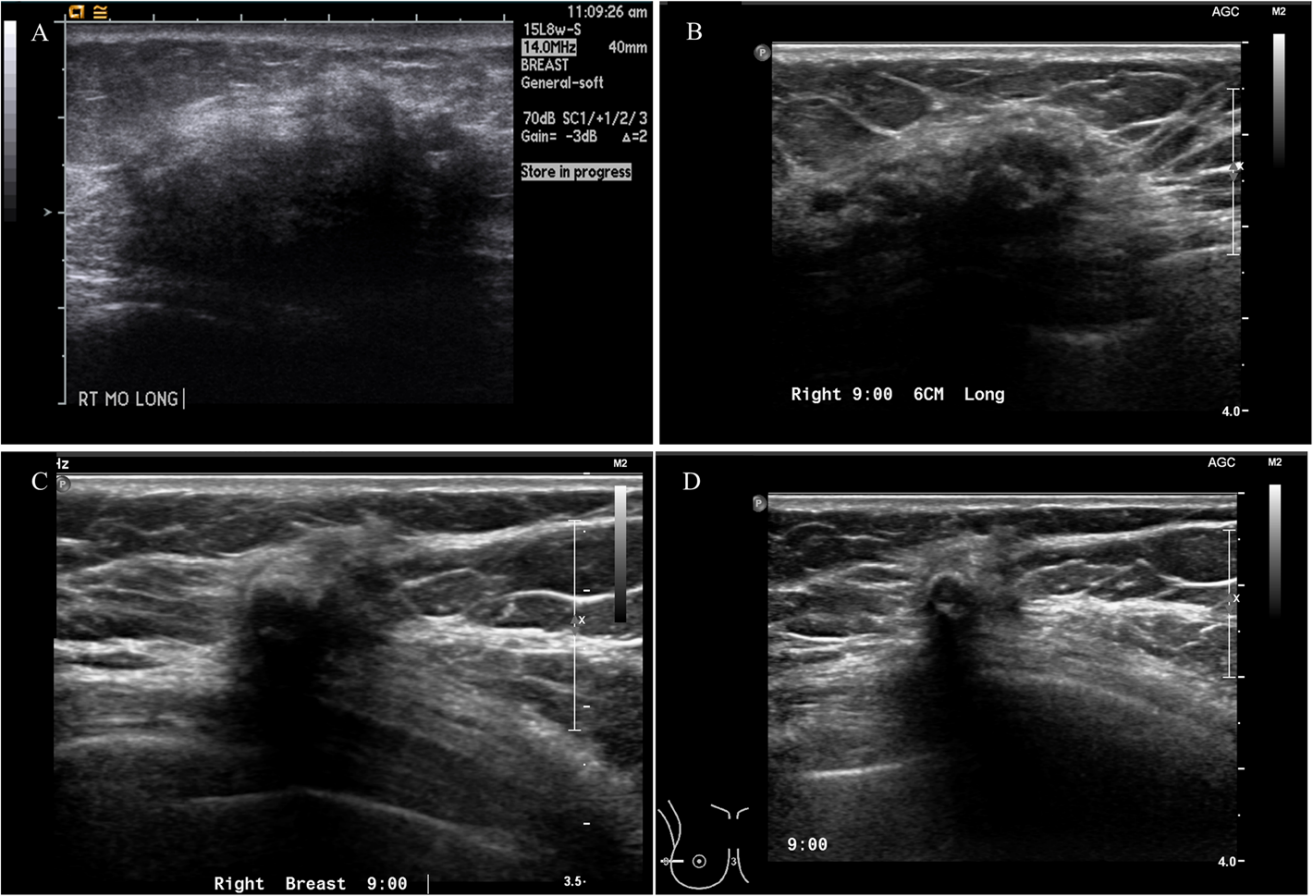
Consecutive changes in fat necrosis after breast reconstruction in ultrasonography. **a** Initially, fat necrosis was detected after 6 months from surgery as a mass of around 4 cm with an indistinct margin in the mid-outer portion of the right breast. Needle biopsy was performed, and fat necrosis was confirmed pathologically. **b** After 1 year from the detection of fat necrosis, the volume of the necrotic mass was decreased. And calcification appeared at the center of the fat necrosis area (arrowhead). **c** After 2 year from the occurrence of fat necrosis, the mass was shrunk further. **d** Although the mass became much smaller over time, calcification (arrowhead) remained at the fat necrosis area

**Fig. 4:**
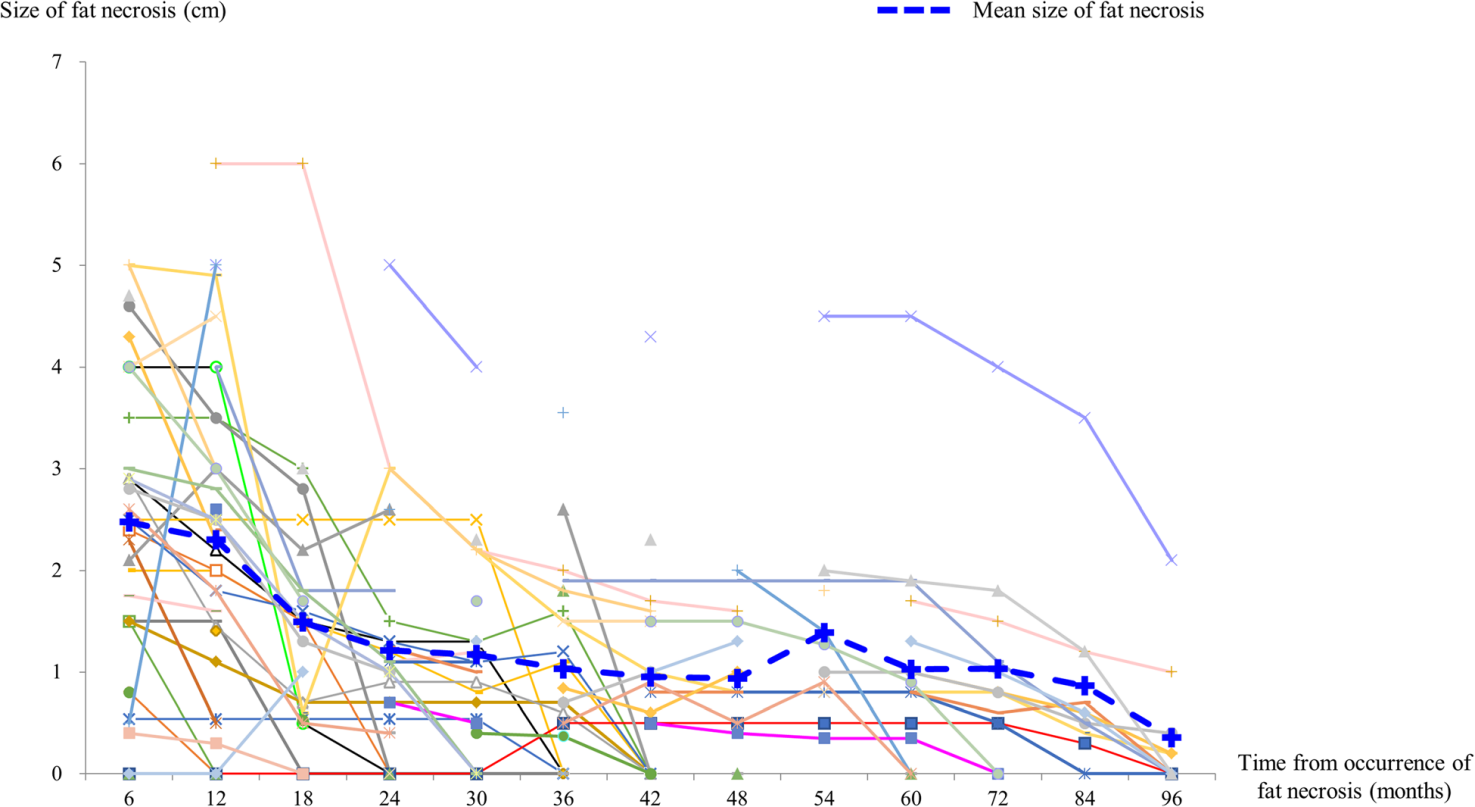
Sizes of the necrotic mass after breast reconstruction. The mean size of the necrotic mass was decreased by half during the first 2 years from the occurrence of fat necrosis, and more than 60% of fat necrosis cases were completely resolved during the 10-year follow-up period

A total of 26 cases (61.9%) of fat necrosis were almost completely resolved during surveillance. The consecutive changes in ultrasonographic findings are shown in Fig. 3, and fat necrosis was resolved after a mean period of 45.9 months (SD, ± 42.1). The mean size of the necrotic nodules was gradually decreased with time, and in most cases, the necrotic nodules were decreased rapidly around 2 years after surgery (Fig. 4).

## Discussion

The rate of breast-conserving surgery among Asian women with breast cancer (50–60%) is relatively lower compared with the rate among American or European patients (70–80%) [15, 16]. This difference may be attributed to the small- to moderate-sized breasts and dense breasts of Asian women (reference). Therefore, the incidence of breast reconstruction after partial or total mastectomy may be higher among Asian breast cancer patients.

Fat necrosis can appear as a postoperative complication after breast reconstruction, and the incidence of fat necrosis after flap-based breast reconstruction is approximately 4–25% of patients treated with breast-conserving surgery and adjuvant radiotherapy for breast cancer [8, 9, 11, 12, 17, 18]. In the current study, the incidence of fat necrosis was 11.6%. Even if fat necrosis is only a minor complication, physicians should carefully evaluate their patients because it is necessary to distinguish it from tumor recurrence [19–21]. And although the adjuvant radiotherapy was applied to ten patients (breast conserving surgery (*n* = 9), mastectomy (*n* = 1)), there was no statistical difference in the incidence of fat necrosis between breast conserving surgery and mastectomy cases. Therefore, the authors suppose that the radiation effect to fat necrosis would be slight.

The identification of fat necrosis can be achieved with clinical examination, mammography, ultrasonography, MRI, or PET/CT [13]. Several studies have reported the imaging findings of fat necrosis using various imaging modalities [8, 17, 18, 22, 23]. Patient who received breast reconstruction for breast cancer can detect a newly formed nodule, including fat necrosis or tumor recurrence. However, because the Asian female patients commonly have high density of breasts, it would be not easy to distinguish between normal parenchyma and newly formed nodule [24].

The mammographic findings of fat necrosis typically show coarsely calcified nodules with occasional lipid-containing complex cysts, increased opacity, microcalcification, or architectural distortion. On the other hand, the ultrasonographic findings of fat necrosis are variable (ranging from a simple or complex cyst to a complex solid nodule). Although solid lesions typically have well-circumscribed margins, they occasionally have indistinct or spiculated margins mimicking carcinoma [18, 25–28]. Cystic lesions appear as complex cysts with a mural nodule, complex cysts with echogenic bands, or round anechoic lipid cysts [29, 30]. Breast MR imaging of fat necrosis also shows a wide range of findings. The internal signal characteristics may be identical to those of the adjacent fat or there may be no enhancement after the administration of contrast material, indicating that the lesion is benign and consistent with fat necrosis. However, contrast enhancement may be present in early periods, complicating efforts to distinguish this entity from recurrent cancer. Enhancement can be focal or diffuse and homogeneous or heterogeneous. Furthermore, enhancement patterns may vary from slow, gradual enhancement to rapid enhancement, and a washout curve may be present [13, 25, 29, 31].

Several surgeons have reported that symptomatic fat necrosis led to a significantly worse cosmetic outcome and that asymptomatic fat necrosis did not demonstrate evidence of cosmetic abnormality [8, 9, 32, 33]. However, they did not observe a significant deterioration in cosmetic outcome for patients who developed fat necrosis. Most cases of fat necrosis may be resolved without intervention or do not show any changes [17, 34]. In this study, more than 60% of fat necrosis cases were spontaneously resolved without any procedures. Around 2 years after the occurrence of fat necrosis, the size of the necrotic nodules was decreased by half and remained constant without significant changes.

According to the literature, the timing of fat necrosis development after breast reconstruction varies widely. Wazer et al. reported the development of fat necrosis as early as 7.5 months [35]. Chen et al. reported fat necrosis at a median time of 66 months [36]. In this study, the mean period until the detection of fat necrosis was 21.1 months from surgery with clinical examination or imaging modalities including mammography, ultrasonography, or breast MR imaging. In addition, the location of fat necrosis after breast reconstruction has been reported to be different based on the surgical techniques used. As fat necrosis is affected by poor blood supply from the breast or flap tissues, the location could be identified with various techniques.

Several risk factors associated with the occurrence of fat necrosis after surgery have been proposed, which include neoadjuvant chemotherapy, adjuvant radiotherapy, high BMI, and uncontrolled underlying disease [37–40]. However, there was no significant factor associated with fat necrosis in our study. The performance of neoadjuvant chemotherapy was only associated with fat necrosis in univariate analysis but not multivariate analysis.

In our study, the radiologist who is an expert in breast with more than 15 years of experiences performed and analyzed about the fat necrosis. If she had been a less experienced radiologist, there would be more cases which requires the pathologic confirmation. In addition, the fat necrosis after breast reconstruction could not be observed as prospective study and this is another limitation in our study.

If the imaging findings of fat necrosis reveal a suspicious lesion, pathologic confirmation should be obtained to rule out tumor recurrence. However, as fat necrosis may resolve independently within 2–3 years, the management of fat necrosis can be performed only through observation if fat necrosis is highly probable or pathologically confirmed. An understanding of the natural course of fat necrosis would be helpful for the management of fat necrosis.

In conclusion, fat necrosis after breast reconstruction for breast cancer is relatively common; nevertheless, it is only a benign complication. Sometimes, it can mimic tumor recurrence, and pathologic confirmation is needed. However, if fat necrosis after breast reconstruction is confirmed, it can only be observed based on its natural course and more than half of the cases will be resolved within 2–3 years.

## Supplementary Information

**Additional file 1.**

## Data Availability

The datasets generated and/or analyzed during the current study are not publicly available due to the data is in company’s possession. But, they are available from the corresponding author on reasonable request.

## Abbreviations

TE: Thoraco-epigastric
LTD: Lateral thoracodorsal
TDAP: Thoracodorsal artery perforator
ICAP: Intercostal artery perforator
LD: Latissimus dorsi
TRAM: Transversus rectus abdominis myocutaneous
DIEP: Deep inferior epigastric
MR: Magnetic resonance
