# Supplementary material for: Natural course of fat necrosis after breast reconstruction: a 10-year follow-up study
Source: BMC Cancer. 2021 Feb 16;21:166. doi: 10.1186/s12885-021-07881-x (PMC7885495; doi:10.1186/s12885-021-07881-x)
Supplement: Supplementary file 1 — Additional file 1. [file 12885_2021_7881_MOESM1_ESM.docx]

Synopsis

Although fat necrosis is a minor postoperative complication after oncoplastic surgery for breast cancer, occasionally it mimics to tumor recurrence. However, based on the natural course of fat necrosis after oncoplastic breast surgery, the fat necrosis after oncoplastic breast surgery can be only monitored, if fat necrosis after oncoplastic breast surgery is confirmed.

Data Availability Statement

Data available on request due to privacy/ethical restrictions.
